# Supplementary material for: Causal Relationship between Aging and Anorexia Nervosa: A White-Matter-Microstructure-Mediated Mendelian Randomization Analysis
Source: Biomedicines. 2024 Aug 16;12(8):1874. doi: 10.3390/biomedicines12081874 (PMC11351342; doi:10.3390/biomedicines12081874)
Supplement: Supplementary file 1 [file biomedicines-12-01874-s001.zip › Supplementary Figure/Figure S4/Singe SNP plot of MR analysis for TL to ubm-b-1567.pdf]

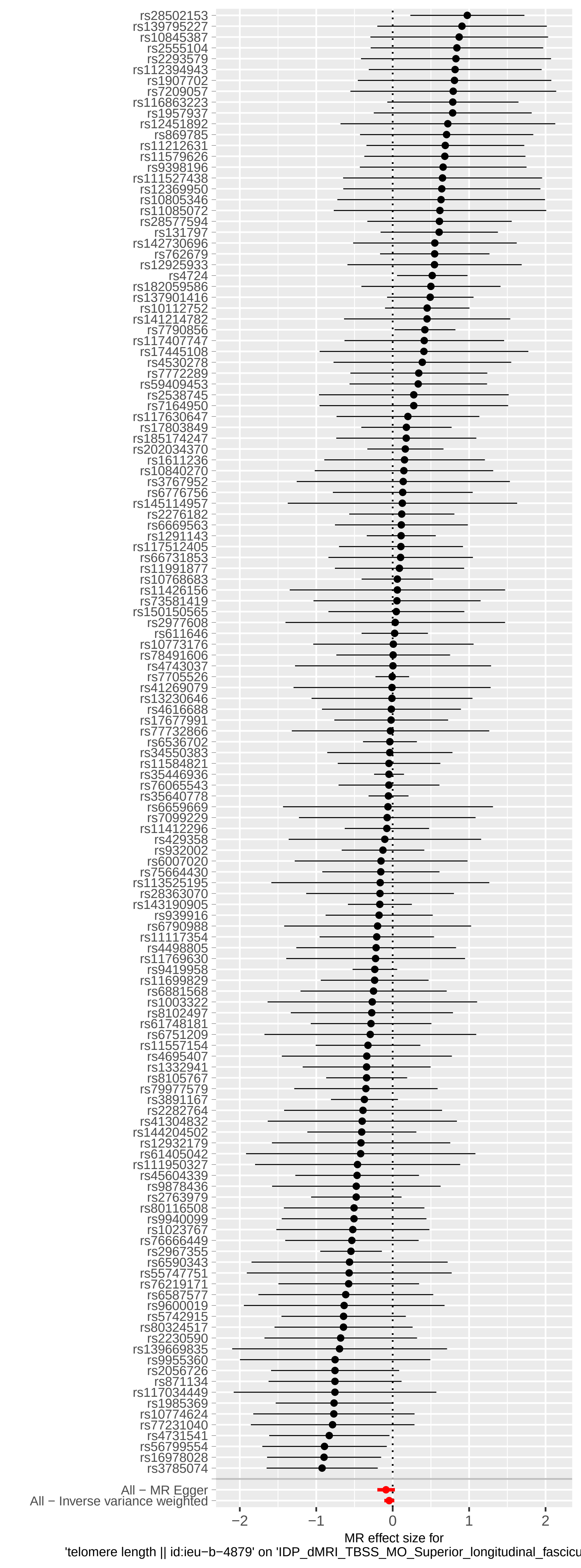
rs28502153
rs139795227
rs10845387
rs2555104
rs2293579
rs112394943
rs1907702
rs7209057
rs116863223
rs1957937
rs12451892
rs869785
rs11212631
rs11579626
rs9398196
rs111527438
rs12369950
rs10805346
rs11085072
rs28577594
rs131797
rs142730696
rs762679
rs12925933
rs4724
rs182059586
rs137901416
rs10112752
rs141214782
rs7790856
rs117407747
rs17445108
rs4530278
rs7772289
rs59409453
rs2538745
rs7164950
rs117630647
rs17803849
rs185174247
rs202034370
rs1611236
rs10840270
rs3767952
rs6776756
rs145114957
rs2276182
rs6669563
rs1291143
rs117512405
rs66731853
rs11991877
rs10768683
rs11426156
rs73581419
rs150150565
rs2977608
rs611646
rs10773176
rs78491606
rs4743037
rs7705526
rs41269079
rs13230646
rs4616688
rs17677991
rs77732866
rs6536702
rs34550383
rs11584821
rs35446936
rs76065543
rs35640778
rs6659669
rs7099229
rs11412296
rs429358
rs932002
rs6007020
rs75664430
rs113525195
rs28363070
rs143190905
rs939916
rs6790988
rs11117354
rs4498805
rs11769630
rs9419958
rs11699829
rs6881568
rs1003322
rs8102497
rs61748181
rs6751209
rs11557154
rs4695407
rs1332941
rs8105767
rs79977579
rs3891167
rs2282764
rs41304832
rs144204502
rs12932179
rs61405042
rs111950327
rs45604339
rs9878436
rs2763979
rs80116508
rs9940099
rs1023767
rs76666449
rs2967355
rs6590343
rs55747751
rs76219171
rs6587577
rs9600019
rs5742915
rs80324517
rs2230590
rs139669835
rs9955360
rs2056726
rs871134
rs117034449
rs1985369
rs10774624
rs77231040
rs4731541
rs56799554
rs16978028
rs3785074
All − MR Egger
All − Inverse variance weighted
−2
−1
0
1
2
MR effect size for
'telomere length || id:ieu−b−4879' on 'IDP_dMRI_TBSS_MO_Superior_longitudinal_fascicu
